# The April Number of the Dental News Letter

**Published:** 1853-07

**Authors:** 


### *The April Number, of the Dental News Letter.*

This comes to us, filled with interesting matter. First, a likeness of Professor White, of the Philadelphia College, of Dental surgery. This gives us a kind of introduction, at least a conception of the man personal, and to some extent intellectual; for we believe in physiognomy. But to aid us in making our estimate, of the intellectual capacity, we have his valedictory address before the first graduating class of the College. This is noticed in a handsome manner, and some good advice given, and then the "forerunning circumstances and influences which brought about the existence of such an instituion." These were first the organization, "of the Pennsylvania Association of Dental Surgeons;" then an application by a few of the members for a college charter, the rejection of this by the Legislature, with the remark of the chairman of the committee of that body, that it was "*inexpedient, impracticable*, unnecessary and without the consent or wish of the majority of the Dentists." This brought out a petition, signed by "*seventy-fiae* of the dentists of the city and state;" also, by the professors of three of the leading medical colleges of the city, as well as by many eminent medical gentlemen in private practice. This gave the legislature to understand, that the profession were in earnest, and had some rights, and a charter was granted.

The Dr. then passes on to the importance of dental education, its connection with general medicine, and makes the following very appropriate remarks.

"Medicine and dentistry are, doubtless, sister professions, but medicine is the older and the larger of the two; and they ought mutually to support each other, and help to push forward the large improvements of the age, so far as they have for their objects the alleviation of human suffering. That medicine is ready to render all possible aid to dentistry, we know to be true; and we here venture to pledge ourselves, in behalf of the faculties of the dental colleges, to supply all demands of medicine so far as our abilities will allow. In view of all this, why heed the caviler in his objections to the course we propose to pursue, or why find fault or indulge in vindicative controversies with our neighbour, if he does not, or cannot, see and believe in the utility of a comparatively new undertaking? With ourselves it is not a matter of life or death, or one in which the question of human right is involved; but merely a difference of opinion as to the best method of accomplishing certain ends in the affairs of business life.

The only question of importance in the matter, gentlemen, is, then, have we been of mutual interest to each other during the course of studies which to-day's proceedings close? If we have, then there is no cause of complaint by the most scrupulous. So far as we are concerned, it has been a constant source of agreeable study and improvement since the course of instructions began; it has required refreshing upon those living principles of our art, which the arduous details of practice are calculated to observe, and required us to make frequent excursions into the beautiful fields of collateral science, to collect such fruits as a large experience in the wants of a practitioner would enable us to bring, to lay before the young inquirer at the shrine of our beloved art. Our reward is ample for all the privation it has cost. If we take the cheerful countenances which have always greeted us when it was our pleasure to meet, and the serious and respectful attention which all were accustomed to give to the various instructions presented for their consideration, and the ability with which each one who was eligible for examination sustained himself, on the several topics on which he was questioned, as a means of judging whether you have not been rewarded, we should most certainly answer, *yes.*

The time has come when our art must be severed from the practice of medicine, in the same manner and on the same principle as the increasing importance and strength of a new colony separates herself from the rule of the mother country, to take rank among the kindred nations of the earth. The science of medicine, and its practice, leaving the study of the teeth out entirely, is already too large to come within the reach of the abilities or health of the mass of students to sustain sufficiently long to fully acquire all that is necessary by the present system of studies. The effort to sustain the grouping together of too many kindred branches of learning, has been sensibly felt in other quarters than our own; hence, the "Brown University," at Providence, Rhode Island, has successfully divided her rooms of study, and classified such branches of learning in each suite as best apply to the wants of the future pursuits of the student."

*Our Profession Abroad.*—We give the two following letters to the News Letter, that our reader may form an idea of our profession abroad. The Americans are becoming missionaries of Dental Science.

My Dear Mr. Jones:—A few days since I received from you, through our mutual friend Mr. West, some excellent tooth-drawing instruments. Please accept my sincere thanks, and I would gladly transfer to you, if it were possible, some of the thanks of the people whom it is often in my power to relieve. You will be glad to know that the instruments are uot useless. Within the first ten or tweve days after their arrival I extracted eight teeth, some of them large, requiring the large foreceps. Many thanks too for the pamphlet. I have derived many useful hints from it. You must know that I am a perfect tyro at the business, having been pressed into the service by sheer necessity. Multitudes of the people, coming from a great distance in some instances, seek from us relief in all surgical and dental cases, which their own physicians never pretend to afford. As a people they are quite careful of their teeth. All classes clean them before eating, rubbing them with a clean twig broken fresh from the tree. They are superstituously exact in this matter, never, as they say, defiling their mouths by putting any thing a second time into them. But, from some cause, their teeth often ulcerate at the roots, which produces a constant discharge through the cheek. On extracting the tooth, the ulcer heals directly. I have relieved a great many such cases. Such teeth are not difficult to draw, often becoming quite loose.

But I need not encroach upon your time by any extended observations, being so incapable of making any that would be of much interest. My principal reason for wishing to do any thing to relieve the people's pains, is the more ready access it gives me to their hearts. A little knowledge of medicine is almost invaluable to a missionary. Their diseases, it often seems to me, are less complicated and more easily cured than those of more civilized people.

Again I thank you for the instruments, and trust your gifts will never be less highly appreciated.

Most truly yours, J. E. Chandler.

Sivagnnga, Southern Indiana, April 2, 1852.

[We take the following extract from a private letter, addressed to our house in Boston, on dentistry in the "Azores."

The method of filling teeth, as described, agrees precisely with the method practsed at Santa Cruz, (one of the West India Islands,) as related to us by an American dentist, who visited there some years since. If we had our choice in such a case, we should prefer extraction.

There is a wide field abroad for American dentists, where fortunes and honors may be secured.—Ed.]

DENTISTRY IN THE AZORES.

Mr. Freeman—*Dear Sir*:—Knowing the interest felt relative to the success of American dentists residing abroad, I take the liberty of addressing you a few lines, trusting they may be acceptable.

You will be surprised when I inform you that, previous to my establishment in the "Azores," the people were almost entirely ignorant on the subject of dentistry; and at Fayal (the first of the group which I visited) they had so little faith in my abilities, that for the first few months I was obliged to content myself by merely displaying my assortment of teeth, instruments, &c.; but after I had inserted a complete set of artificial teeth for an English lady, I succeeded in obtaining the confidence of the public, and they were then so anxious to avail themselves of my services, that I was allowed to practice without passing the examination required by law.

My method of inserting teeth, from one to a whole set, without springs or clasps, astonished them exceedingly; and when they witnessed some of my large gold plugs, in teeth which they considered impossible to preserve, and which I had been requested to extract, their admiration was increased.

Through the influence of the physicians, I was enabled to introduce the use of chloroform and ether, for the alleviation of pain during the extraction of teeth ; and as they were almost always present at such times, I very soon convinced them of the safety of anæsthetic agents when properly applied; so much so that they made use of them in the hospital and their private practice.

When I came to this city I found the people very timid in regard to dental operations, and notwithstanding I brought the very best of recommendation, I had considerable trouble in obtaining the favor of the public, who were prejudiced against me, owing to the sayings and doings of a Frenchman, who calls himself a dentist,(?) and says he studied with one of the first professors in Paris. Dentistry must be in a primitive state in Europe, particularly in France, if I may judge from some specimens of his production. Why, an American student of a month's experience would be ashamed if he could not do better. I think I shall succeed in securing one of the specimens; if so, I will send to you one as a curiosity.

His manner of plugging teeth is certainly original. As yet I have heard of but one person who was able to bear the operation. A man applied to him to have one of his inferior molars plugged; the cavity was prepared, and a piece of lead or tin, (perhaps fusible metal,) of the size required, was placed in the tooth, a hot iron introduced into the mouth, and the substance melted into the cavity, causing such intense suffering that it can be better imagined than described.

He knows nothing about inserting teeth by means of atmospheric pressure, and actually declares that it cannot be done, although I have inserted several sets, to the entire satisfaction of the patients.

My success in curing diseases of the gums has created no little surprise, and many of the physicians are so much pleased with my mode of treatment, that they now send their patients to me when there is any derangement of the dental organs.

I might relate several interesting incidents connected with my practice in these islands, but I must defer it to another time.

Very truly yours, W. C. Starbuck, Jr.

Cidade de Ponta Delgada, San Miguel, Oct. 20, 1852.

### *Artificial Lips and Noses.*

It is presumed that editors should know every thing. The letter to Messrs. Jones, White and McCurdy is an evidence of this, and reminds us of one of a like character, only rather more professional. We should certainly refer such correspondents to some of our plastic operators. Professor Mussy of this city, we know could make a nose in this way, but for the lip I doubt if it would do to kiss. An ivory lip might look well enough, but there would not be much nectar about it. But we give the letter.

Messrs. Jones, White & McCurdy:—Having been applied to by a gentleman for a job of work, I take the privilege of making a few inquiries concernrng it. The case is this: The gentleman wants an artificial nose, lip, and a set of artificial teeth. I would like to know from you what an artificial nose and lip would cost. I can answer about the teeth.

I would also like to have some information about the best manner of putting up the job. If you can give me any information, I would be very thankful. I have never seen any thing of the kind put up, and am at a loss in what way to do it. I would like very much to do the work is the reason of my making the inquiry I do. Yours, &c.

### *Impracticable Theories.*

This is the heading of a few criticisms on the new operation of risodontropy, by M. M. Frisselle M. D., in the Boston Medical and Surgical Journal. Some of the remarks are very appropriate and deserve notice, others show not as much anatomical knowledge as we should expect from a dentist. We suppose if the nerve is entirely severed, with its investing membrane, that reunion would not always take place. A complete separation is not necessary, and we suppose is not usually accomplished, and we see no physiological reason why the cut nerve may not unite and give us a vital organ. A tooth deprived of its vitality by this operation, is no better than if deprived of the nerve by excission, through the carious opening. A drill to sever nerve and membrane must be as large as the dental foramen, and this is larger than is recommended. A large majority of the bicuspids of the superior maxilla have two nerves, corresponding to the outer and inner cusps of the teeth. 'Tis true, a large majority of these teeth have but one root, yet this is a flattened one, representing two roots united. It often appears as if the two nerves were enveloped in one membrane, yet every practical dentist must have observed that after the one is excised, the vitality of the other exists. We are aware that Dr. Miller recommends the entire severing of the nerves, because they are not so susceptable to impressions of heat or cold. We have repeatedly performed the operation, and excepting in a few cases, have not completely severed the nerve, but most frequently could we detect vitality in the pulp through the carious opening. Not enough it is true to interfere with the operation of filling. And excepting in one or two instances, we have been able to keep down inflammation, and save the organ and also its vitality. The last number of the same journal is received, and contains another article on the same subject, which we may notice in the next number of the Register.

We prefer our readers should see the opinion of our medical brethern on this operation, and give the following remarks from Dr. Frisselle's article.

"The surgical operation which has of late clalmed the attention of the medical profession—called "Hullihen's operation," or Miller's operation (as may best please)—has excited considerable interest, and I trust may be produtive of much good; but in order to judge correctly of the practability of any qnestion, or practice, the evils as well as the benefits growing out of it should be considered. The advocates of the new operation claim, I believe, that the surgeon is enabled to plug the carious tooth when the dental pulp is exposed, without pain, and preserve the vitality of the tooth. I do not quite understand how the nerves and blood-vessels can be severed, and, as in some cases stated, the dental pulp removed, without destroying the vitality of the organ. It has always been my impression that when the circulation of the nervous communication is cut off from an organ, it immediately looses its vitality, and nature soon makes effort to remove it. I cannot see why the same rule does not apply to the teeth. So far as my observation goes, it does; for when the vitality of a tooth is destroyed, nature makes effort to rid herself of it, and will do so sooner or later, either by ulceration taking place around the fang, or by absorption. Then, if the vitality of the tooth be destroyed by Miller's operation, (or Hullihen's,) the organ can be in no better condition than if it had been destroyed through the carious cavity; and the only advantage gained is, that it is more convenient, and an opening is left for the discharge of pus, should any be formed. It is stated by one of the suggestors of this operation, that when the dental pulp was removed, the result "in every case, so far as known, has been as successful as when the pulp was allowed to ramain" also, "that when the pulp is removed, the teeth are not sensible to impressions from heat and cold." Now, if the dental pulp can be removed by ulceration or by mechanical means, and the tooth still retain its vitality, and not be sensible to impressions from heat and cold, I must confess that the dental surgeon has made a discovery which must entirely revolutionize all our previous notions of vitality as dependent upon the nervous system and the circulation.

Dr. Miller, in the second of his reported cases found in the Journal, vol. xlvii., says, "having amputated the nerve of the cupidates, a query arose as to what should be done with the bicuspids having *two* nerves. After a moment's reflection the drill was carried deeper, cutting off both branches, and the teeth filled without pain." If the doctor had reflected two minutes, instead of one, he might have come to the conclusion that the bicuspids are furnished with but *one* nerve, instead of two, as a general rule—those that have two (as is sometimes the case) being *exceptions*, and not the rule.

